# Evaluation of clinical and radiographic warning signs for prediction of oroantral communication following tooth extractions

**DOI:** 10.1007/s00784-024-06000-x

**Published:** 2024-10-23

**Authors:** Alexandra Jurásek, Nelli Farkas, Dorottya Frank, Béla Kolarovszki, Balázs Sándor, Andrea Radácsi, Ildikó Szántó, Krisztián Katona

**Affiliations:** 1 Department of Dentistry, Oral and Maxillofacial Surgery, University of Pécs, Medical School, Tüzér Street. 1, 7623 Pécs, Hungary; 2 Institute of Bioanalysis, University of Pécs, Medical School, Pécs, Hungary

**Keywords:** Maxillary Sinus, Tooth Extraction, Intraoperative Complications, Oroantral Fistula, Orthopantomography, Panoramic radiograph

## Abstract

**Objectives:**

Oroantral communication (OAC) is a relatively common and mild complication of maxillary tooth extractions. Preoperative prediction of OAC can reduce treatment duration and prepare both operators and patients for the procedure. This study aims to identify alarming radiographic and clinical indicators that can predict OAC therefore assisting clinical decision making to practicing general dentists.

**Methods:**

In this retrospective case–control study the OAC group consisting of 97 cases and a control group twice the size was established. Clinical data were collected, and measurements were conducted separately by two blinded observers on digital panoramic radiographs. Inter-rater reliability was assessed. In case of disagreement a third observer’s results were utilized. The correlation between OAC and demographic data (age, sex), as well as various factors assessed on panoramic radiographs (including, but not limited to, the length of the root, root projection into the sinus, bone width, presence of mesial and distal adjacent teeth), was statistically evaluated.

**Results:**

Inter-rater reliability was found to be excellent. Several factors were identified as potential predictors of OAC. According to our model, the strongest predictors were the distance between the cemento-enamel junction and marginal bone, extent of root projection into the sinus, presence of sinus recess around the roots, angulation, and absence of the mesial adjacent tooth.

**Conclusions:**

Well-defined measurements on panoramic radiographs may aid in predicting OAC. Further prospective investigations are necessary to confirm these indicators and address factors related to clinical examination and operation.

**Clinical relevance:**

We present several clinical and radiographic warning signs of OAC that can facilitate pre-extraction decision-making.

**Supplementary information:**

The online version contains supplementary material available at 10.1007/s00784-024-06000-x.

## Introduction

Oroantral communication (OAC) is a relatively common complication of maxillary tooth extractions[1–3] and other interventions such as sinus lifts and implant placement [4]. The highest incidence of OAC is reported in cases of maxillary first and second molar extractions [1], although canine and premolar extractions may also result in OAC [5]. Well-known risk factors predisposing individuals to this complication include the extraction of the last remaining tooth from the arch or a tooth with periapical inflammation [1, 4, 6]. Morphometric studies have highlighted the importance of the size, shape, and extent of the maxillary sinus, as well as the relationship between the roots of maxillary teeth and the floor of the sinus [7–17]. These works provide essential insights into the anatomical variability of the maxillary sinus and highlight the benefits of analysing cone beam computed tomography (CBCT) over panoramic radiography (PR) [7, 10–12, 14, 17, 18]. However, these studies do not provide clinical guidance. Potential risk factors of OAC were examined most broadly in relation to upper wisdom tooth removal [18–22] and mostly reported the superiority of CBCT analysis over panoramic radiographs in the prediction. While cone beam computed tomography (CBCT) has been shown to provide valuable insights into these anatomical considerations, panoramic radiographs remain a more commonly selected diagnostic tool due to their lower cost and reduced radiation exposure [23]. [24] Unlike in case of impaction, OAC during routinely performed extractions can significantly extend the treatment duration [25], as it will require additional interventions such as raising a flap, which can lead to further potential complications [5].

Our study aims to assess and identify potential clinical and panoramic radiographic warning signs that could predict the occurrence of OAC during maxillary tooth extractions. Such risk assessment could benefit both patients and dentists by improving time management, aiding in decision-making, enhancing informed consent processes, and facilitating treatment planning for more favourable long-term outcomes.

## Materials and methods

In our retrospective case–control study, approved by the regional ethics committee (8577-PTE 2020), a database search was conducted in the archives of the University of Pécs, Clinical Centre, Department of Dentistry, Oral and Maxillofacial Surgery. All cases in which closure of OAC was performed between 2019 and 2021 were collected. The cases were then categorised based on the inclusion and exclusion criteria outlined in Table 1. Two controls were matched to each OAC case solely based on the type of tooth (canine (C), first premolar (PM1), second premolar (PM2), first/second or third molar (M1/M2/M3)). A written database was employed to document the interventions performed, specifically focusing on the extractions of upper canines, premolars, and molars that did not result in OAC. For each potential candidate identified for inclusion, we verified the availability of appropriate imaging prior to the extraction and ensured that the patient satisfied all inclusion criteria while adhering to the exclusion criteria. Candidates meeting all specified criteria were included in the study. This process continued until each case was matched with two suitable controls.

**Table 1:** Inclusion and exclusion criteria (OAC-oroantral communication, PR- panoramic radiographical image, C-canine, PM1/PM2- first or second premolar, M1/M2/M3- first, second or third molar)

*Inclusion criteria- case*	*Exclusion criteria- case*
• OAC after tooth extraction of a maxillary canine, premolar or molar tooth	• OAC was not the result of tooth extraction, • tooth extraction was not performed at University of Pécs, Clinical Centre, Department of Dentistry, Oral and Maxillofacial Surgery (referred patients), • lack of PR prior to extraction, • PR was older than 6 months or surgical interventions (extractions, implantation) were carried out in the quadrant within the time span between the PR was taken and the OAC developed, • multiple extractions were performed in the same quadrant (localisation of OAC and relationship to adjacent extraction was unknown), • uninterpretable PR
*Inclusion criteria- control*	*Exclusion criteria- control*
• 2 controls for each case with the same type of extraction (C, PM1, PM2, M1/M2/M3) without OAC after extraction	• no prior PR, • uninterpretable (blurry) PR, • multiple extractions from the same quadrant

**Fig. 1:**
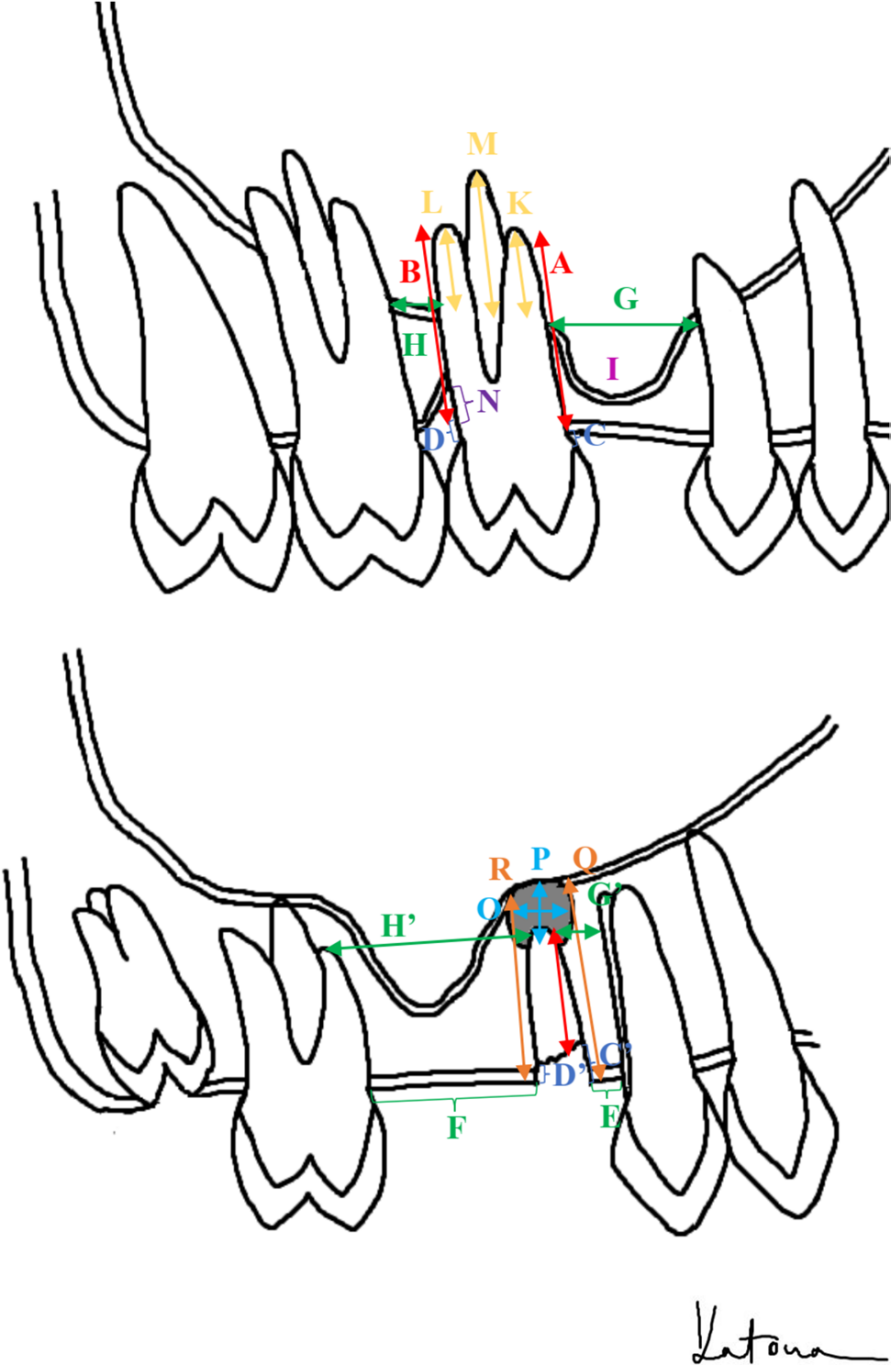
Schematic representation of the measurements conducted on the panoramic radiographical images: *A* and *B*- length of the root measured mesially and distally from the coronal level of the bone to the apex; *C* and *D*- distance between the cemento-enamel junction (CEJ) and the coronal bone level on the mesial (*C*) and distal (*D*) sides. In cases of crown restoration, the most apical point of the crown was used instead of the CEJ. For severely destructed teeth (root remnant) below the bone level (*C*', *D*') or in cases of impacted teeth, this value is negative; *E* and* F*- interdental space measured coronally on the mesial and distal sides, at the coronal bone level or, in the case of an impacted tooth, at the most coronal point of the crown. If no more tooth was present in the quadrant, the distal measurement point was designated at the distal side of the maxillary tuber and the mesial at the median sagittal suture; *G* and *H*- interdental space measured apically on the mesial and distal sides at the level of the sinus base, or if the root did not reach this, then at the most apical point of the root (*G*' & *H*'). In cases of completely missing distal or mesial teeth in the quadrant, the same approach was used as previously described; *I*-sinus recess on the mesial side (or the distal side); *K* and *L*-root projection into the sinus, on the mesial and/or distal side: distance between the level of the sinus base and the apex on the mesial and distal side, note that if the apex does not reach the level of the sinus base this value is negative; *M*- maximal root projection- the length between the base of the sinus and the apex of the root with the most protrusion into the sinus. If the root(s) do not reach the base of the sinus, the distance between the base and the closest root was measured and indicated by a negative value; *N*-depth of vertical bone loss: distance between coronal level of bone and most apical point of vertical defect; *O* and *P*- mesiodistal (*O*) and vertical (*P*) diameter of periapical defect; *Q* and *R*- vertical bone width on the mesial and distal sides, measuring the distance between the coronal bone level and the base of the sinus on the mesial and distal sides of the analyzed tooth, respectively

Two independent interpreters (dentists with 3 + years of clinical experience), blinded to the case/control status, evaluated the panoramic radiographs according to a predefined criteria system (see Appendix 1). In the initial phase of the study, five panoramic radiographs from the clinic's database, which were not included in the analysis (not OAC cases), were selected for the purpose of practicing measurements and reaching a consensus on the precise methodology for evaluating each parameter. Following this training, the observers conducted their assessments independently. All X-rays were captured at the university clinic using the same X-ray machine (VATECH, PCH-2500, Korea) and following the manufacturer’s recommended positioning for each patient. The panoramic radiographs were assessed using EasyDentV4 (Vatech Co., Ltd., version: 4.1.3.2, Data Tec, Inc., Johannesburg, South Africa) software, that allows measuring based on average distortion (“calibration by model”). The linear measurements conducted on the images are depicted in Fig. 1. Scores for bivalent features such as the presence of filling, caries, and periapical radiolucency were evaluated as present (1) or absent (0) by each observer.

### Statistical analysis

The interrater reliability was determined using Cohen's Kappa. In cases where discrete variables did not align between the two interpreters, the results of a third interpreter's evaluation were utilised for further analysis. The third observer was trained to evaluate on the same aforementioned PR images. Similarly, the results of the third interpreter were considered in instances where the interrater κ was < 0.8 (indicating less than strong agreement) for continuous variables. All three observers had more than 3 years of clinical experience as a dentist.

Exploratory analyses were conducted using Wilcoxon rank sum tests, Pearson’s Chi-squared tests, and Fisher’s exact tests. Given the high number of variables relative to the sample size, a random forest algorithm was employed for variable selection to identify potential predictor factors for OAC. Variable correlation for continuous variables was assessed to identify correlated and independent variables. A model comprising only independent factors potentially influencing OAC was developed based on the outcomes of the variable selection and correlation. Decision tree and binary logistic regression analyses were performed using this model.

## Results

**Fig. 2:**
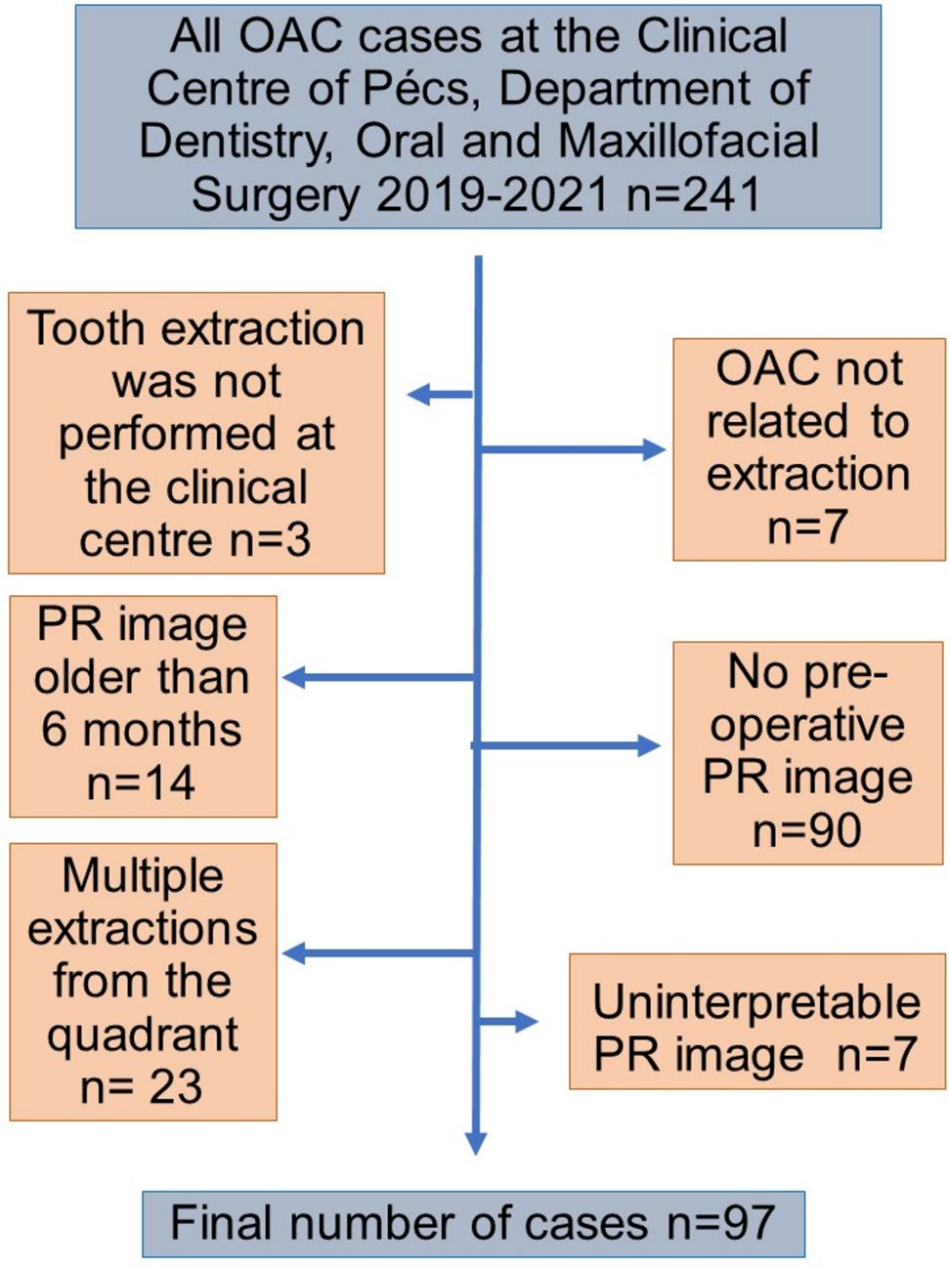
Flow chart of case selection, OAC-oroantral communication, PR-panoramic radiographic (image)

A total of 241 cases of oroantral communication (OAC) were evaluated, of which 97 cases were ultimately included in the study. The process of case selection is depicted in Fig. 2.

The selected cases and controls, as well as the demographic data (age, sex) of the patients and factors related to tooth extraction (tooth position), were collated in a single table (Table 2).

**Table 2:** Demographic and clinical data of the study population, C-canine, PM-premolar, M-molar

	*Case*	*Control*
n	97	194
Female	54	99
Male	43	95
Average age (years)	43 ± 18	42 ± 18
C	2	4
PM1	3	6
PM2	7	14
M1	46	92
M2	26	52
M3	13	26

No significant difference was found between the case and control groups regarding age (*p* = 0.689) and sex (*p* = 0.455).

In the case of all categorical variables, there was a near-perfect agreement between the interpreters (κ > 0.81). In instances of disagreement, a third independent interpreter's decision was utilized for further assessment. The only exception was the measurement of the vertical width of the periapical defect, which demonstrated less than perfect agreement (κ = 0.68).

Among the factors examined, several were identified as potential predictors of OAC. A statistically significant correlation was observed with impaction (*p* = 0.001), the distance from the cementoenamel junction (CEJ) to the marginal bone on the distal (*p* = 0.002) and mesial sides (*p* = 0.002), maximal root projection mesially (*p* < 0.001) and distally (p = 0.001), and the vertical bone width mesially (*p* = 0.002) and distally (*p* = 0.009) (Table 3).

**Table 3:** Results of Wilcoxon rank sum tests, Pearson’s Chi-squared tests and Fisher’s exact tests CEJ^*^-cemento-enamel junction, SD^**^-standard deviation, V^***^-vertical, M^****^-mesial, D^*****^-distal. *Italic* p values are significant. All measured parameters given in millimetre

	*Case*	*Control*	*p*
Maximal root projection (average ± SD)	2.9 ± 3.5	1.1 ± 3.0	< *0.001*
Distance between CEJ^*^ and marginal bone on the distal side (average ± SD^**^)	2.19 ± 2.80	3.39 ± 2.15	*0.002*
Distance between CEJ and marginal bone on the mesial side (average ± SD)	1.71 ± 2.83	3.04 ± 2.05	*0.002*
Vertical bone width distally (average ± SD)	6.6 ± 3.1	7.5 ± 3.1	*0.009*
Maximal root projection mesially (average ± SD)	1.46 ± 3.03	0.05 ± 2.83	< *0.001*
Maximal root projection distally (average ± SD)	-0.20 ± 2.63	0.99 ± 2.62	*0.001*
Vertical bone width mesially (average ± SD)	6.4 ± 3.1	7.8 ± 3.6	*0.002*
Distal adjacent tooth present (average ± SD)	43	95	0.455
Mesial adjacent tooth present (average ± SD)	53	121	0.205
Length of root mesially (average ± SD^)^	8.90 ± 3.06	8.49 ± 2.55	0.358
Caries present	74	164	0.086
Distal sinus recess present	8	8	0.146
Angulation	V^***^:65 M^****^:21 D^*****^:11	142 35 17	0.540
Root canal treated tooth	20	29	0.223
Interdental space distally coronally (average ± SD)	6.50 ± 6.40	5.70 ± 6.70	0.175
Interdental space mesially apically(average ± SD)	9.00 ± 10.00	7.00 ± 7.00	0.271
Periapical lesion reaches/penetrates the base of the sinus	28	60	0.718
Interruption in the basal line of maxillary sinus	13	17	0.220
Interdental space mesially coronally (average ± SD)	7.00 ± 10.00	5.00 ± 7.00	0.197
Periapical bone resorption present	30	73	0.260
Interdental space distally apically(average ± SD)	7.20 ± 6.60	6.80 ± 6.90	0.498
Maximal mesio-distal width of periapical defect (average ± SD)	1.92 ± 3.14	1.91 ± 2.81	0.588
Multiple roots	80	165	0.570
Mesial sinus recess present	19	25	0.133
Impaction	6	0	*0.001*
Length of root distally (average ± SD)	8.05 ± 2.82	7.56 ± 2.68	0.211
Maximal depth of vertical bone defect (average ± SD)	0.48 ± 1.20	0.58 ± 1.28	0.733
Restoration in tooth present	36	70	0.863
Relation of apex(es) to base of maxillary sinus	root projects into the sinus: 67 in contact: 13 no contact: 17	122 26 46	0.467
Vertical bone loss present	20	41	0.919
Maximal vertical width of periapical defect (average ± SD)	0.77 ± 1.57	0.76 ± 1.49	0.532
Interdental space distally coronally (average ± SD)	6.50 ± 6.40	5.70 ± 6.70	0.175
Vertical bone width mesially (average ± SD)	6.4 ± 3.1	7.8 ± 3.6	*0.002*

**Fig. 3:**
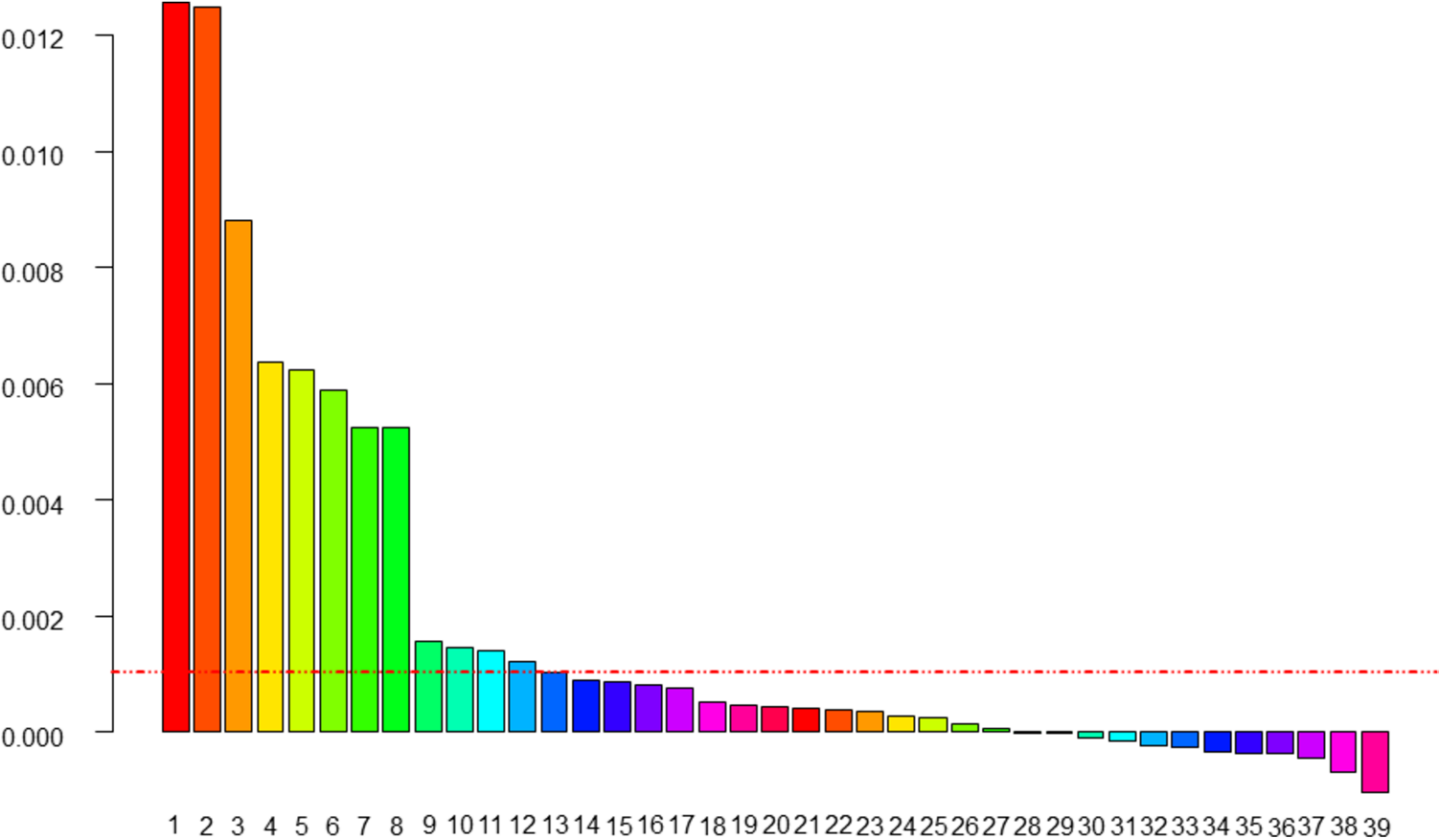
Results of random forest analysis. Each column represents a variable (1–39), factors above the line (1–13) were identified as potential predictors of oroantral communication: *1*- maximal and *2*-average root projection, *3*- distance between cementoenamel junction and marginal bone on the distal and *4*- mesial side, *5*-vertical bone width distally, *6*- maximal root projection mesially, *7*- maximal root projection distally, *8*- vertical bone width mesially, *9***-**presence of distal adjacent tooth, *10*- extent of caries lesion, *11*- presence of mesial adjacent tooth, *12*-length of root mesially, *13***-**presence of caries, *14*-distal sinus recess present, *15***-**angulation, *16***-**presence of sinus recess, *17***-**root canal treatment (yes/no), *18*-interdental space distally coronally, *19*-age of patient (years), *20***-**interdental space mesially apically, *21***-** relation of periapical lesion to the base of the sinus, *22***-** interruption in the basal line of maxillary sinus, *23***-**interdental space mesially coronally, *24***-**presence of periapical radiolucency, *25*- interdental space distally apically, *26***-** maximal mesio-distal width of periapical defect, *27***-**single or multiple roots, *28***-**presence of mesial sinus recess, *29*-impaction, *30*-root length distally, *31*-depth of vertical bone defect, *32*-type of tooth, *33*-presence of restoration, *34*- extent of restoration, *35*-relation of apex(es) to base of maxillary sinus, *36*-sex, *37*-presence of vertical bone defect, *38*-maximal vertical width of periapical defect, *39*-notation of tooth (FDI)

A random forest analysis for variable selection identified 13 factors potentially related to OAC (Fig. 3).

**Fig. 4:**
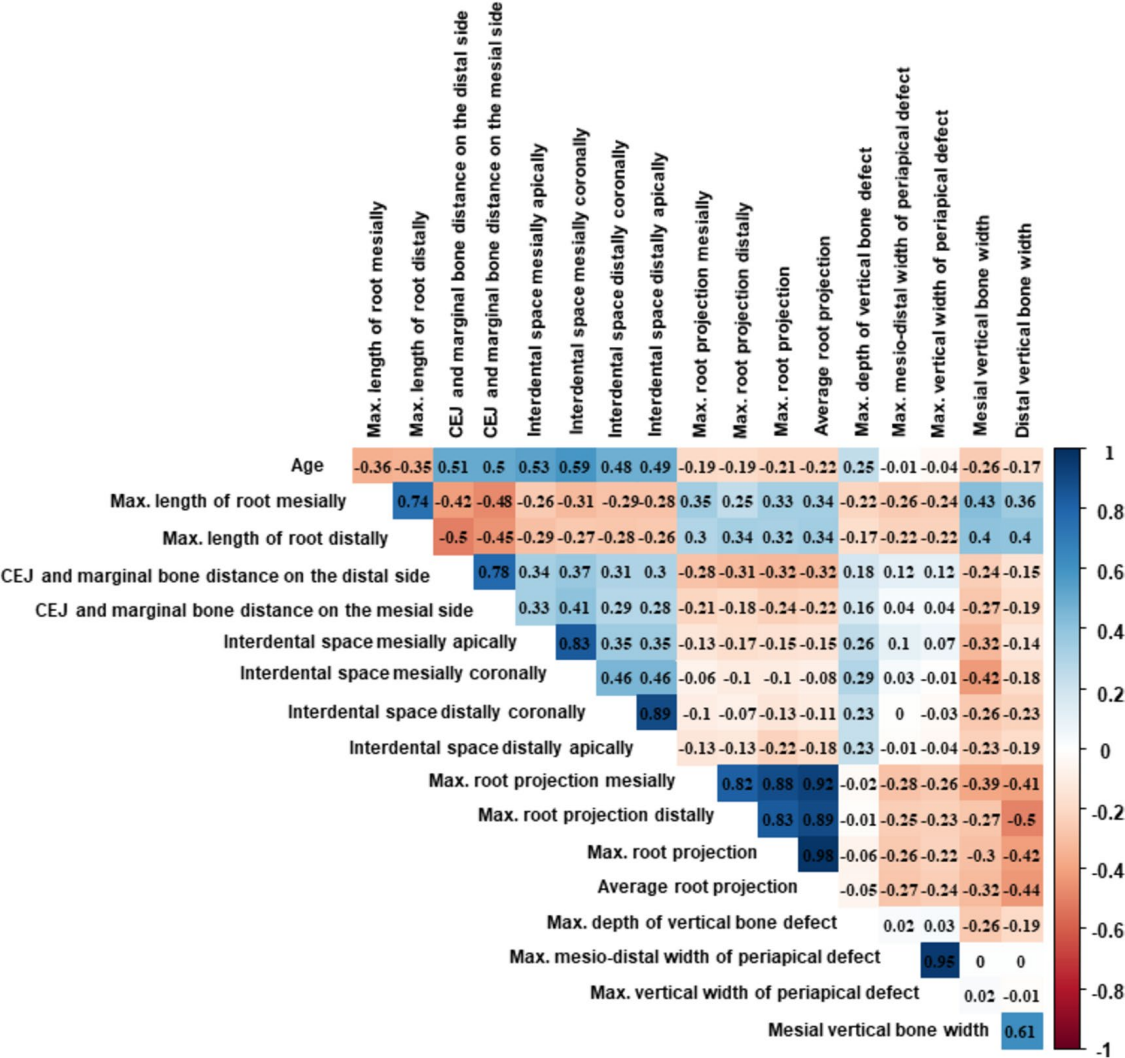
Results of variable correlation: values closer to 0 indicate no correlation while values closer to 1 or -1 indicate strong positive and negative correlation respectively. CEJ-cemento-enamel junction, max.-maximal

As anticipated, the correlation of variables revealed several interacting factors (Fig. 4).

A model based on the gathered information was constructed and utilized for further evaluation. Binary logistic regression analysis revealed three factors that significantly influenced OAC: maximal root projection (*p* < 0.001; odds ratio [OR] = 1.22), the distance from the CEJ or the most coronal portion of the root to the marginal bone on the mesial side (*p* = 0.011, OR = 0.721), and the presence of an adjacent mesial tooth (*p *= 0.032, OR = 0.495) (Table 4).

**Table 4:** Results of binary logistic regression analysis. *Italic* p-values indicate significant correlation. OR-odds ratio, CI-confidence interval, CEJ-cemento-enamel junction

*Variable*	*OR*	*95% CI*	*p-value*
maximal root projection	1.22	1.11, 1.35	< *0.001*
distance between CEJ and marginal bone on the distal side	1.02	0.802, 1.28	0.899
distance between CEJ and marginal bone on the mesial side	0.721	0.555, 0.923	*0.011*
adjacent tooth mesially			
not present	1.00	–	
present	0.495	0.257, 0.938	*0.032*
adjacent tooth distally			
not present	1.00	–	
present	1.05	0.454, 2.48	0.905
presence of caries			
not present	1.00	–	
present	0.613	0.299, 1.27	0.183
interdental space distally coronally	1.02	0.959, 1.09	0.492
maximal length of root mesially	0.919	0.811, 1.04	0.176
presence of sinus recess			
not present	1.00	–	
present	1.80	0.918, 3.50	0.085
angulation			
vertical	1.00	–	
mesial	0.956	0.453, 1.98	0.905
distal	1.45	0.489, 4.15	0.492

**Fig. 5:**
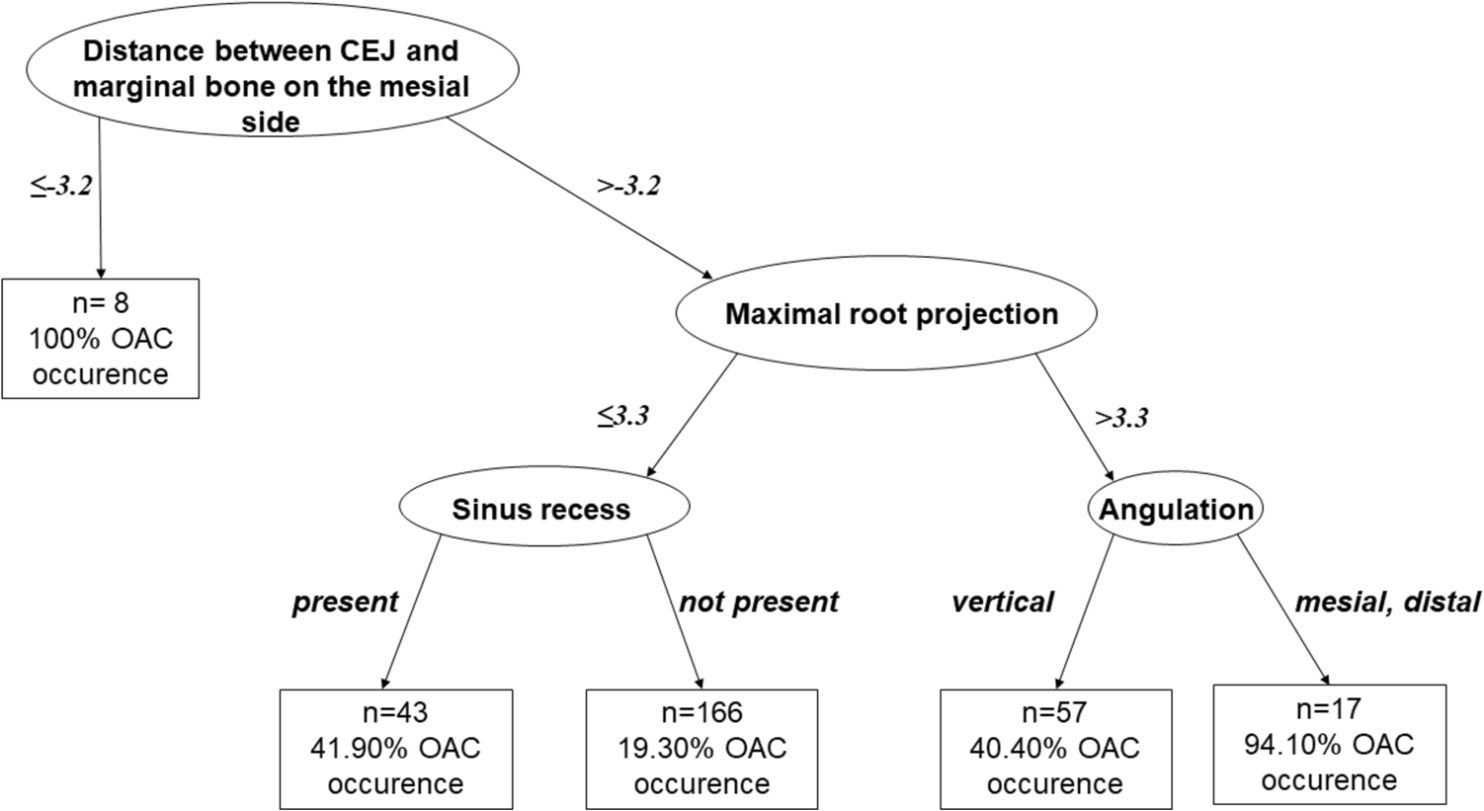
Decision tree for oroantral communication occurrence

According to the decision tree analysis, four branching factors were identified: distance from the CEJ or the most coronal portion of the root to the bone level on the mesial side, maximal root projection, presence of sinus recess, and angulation (Fig. 5.).

## Discussion

OAC associated with maxillary tooth extractions is a relatively common complication. Both the planning and timely intervention in the treatment of OAC can pose challenges for clinicians. Panoramic radiographs are commonly used for the prediction of inferior alveolar nerve exposure during wisdom tooth surgeries [26–28], failure of condylar neck osteosynthesis[29], impaction of maxillary canines [30], growth changes associated with orthodontic therapy [31] and the diagnosis of atheromatous plaque formation in the carotid artery [32]. Panoramic radiographs are generally accepted as diagnostic tools and, in many cases, provide sufficiently reliable measurements [23]. The relationship between the roots of maxillary teeth and the sinus has been evaluated using both panoramic radiographs and CBCT scans [7–10, 12–14, 16, 17]. Although most studies emphasize the superiority of CBCT over panoramic radiographs in assessing the real correlation between the roots and the sinus floor, they do not provide a prediction of the occurrence of post-extraction OAC. Regnstrand et al. reported that approximately 70% of upper first molar roots are in contact with the sinus, with up to a fifth of the root surface (for the palatal root) being involved [8]. However, Punwutikorn et al. found that the incidence of OAC related to upper first molar extractions was only 0.61% [1] suggesting that anatomical observations do not directly translate to clinical findings. According to Sharan & Madjar, the projection of maxillary teeth roots into the sinus is overestimated on panoramic radiographs in both occurrence and length [12]. Jung & Cho reported that, contrary to the appearance of wisdom tooth roots projecting into the sinus on panoramic radiographs, CBCT scans showed that the sinus floor is often located buccally to the roots, mimicking root projection on panoramic images [17]. The risk of OAC during upper wisdom tooth removal has been examined in detail by several studies [18–22], both panoramic radiographs and CBCTs were evaluated, along with clinical parameters. Iwata et al. concluded that the usefulness of computed tomography evaluation as an adjunct to panoramic radiographs in predicting OAC following upper wisdom tooth removal is limited [19]. In addition to root projection into the sinus and depth of impaction, other factors such as a single-rooted tooth, pericoronitis, and "remarkable hemorrhage" were associated with an increased risk of OAC. Hasegawa et al. [20] reported similar outcomes related to the depth of impaction and the root projection, additionally mesioangular position and incision were raised as risk factors. Further risk factors, such as older age and intraoperative root fracture, were reported by Rothamel et al.[22]. A systemic review by Lewusz-Butkiewicz et al.[6] concluded that the relationship between the root of the wisdom tooth and the maxillary sinus can be an important predictive factor, along with older age, mesioangular position, and performed osteotomy during tooth removal. Our study was not conducted focusing solely on wisdom teeth, as the occurrence of OAC is more common and may be a more troublesome consequence when extracting other teeth. Similarly to these articles, "depth," represented in our study by the distance between the cementoenamel junction and the bone margin, was an important predicting factor for OAC. In cases of impaction and severely destructed teeth with remaining roots below the marginal bone level, negative values of this parameter indicated a higher chance for the formation of an oroantral communication. Angulation other than vertical and the length of root projection into the sinus (maximal root projection) also proved to be significant. Unfortunately, in our retrospective study, operational parameters such as excessive use of an elevator, osteotomy, and excessive bleeding were difficult to address; however, they could have had a remarkable impact. As we examined OAC related to the extractions of canines, premolars, and molars as well, the number of cases is higher (97 OAC) than in those studies that focus on wisdom tooth surgeries (7–46), except for a prospective multicenter study by Rothamel et al.[22].

In a recent study by Vollmer et al.[33], several deep learning models were employed to determine if OAC could be predicted based on preoperative panoramic radiographs. They assessed both expert performance and artificial intelligence (AI) performance in predicting OAC without a preset criteria system. From the 100 OAC cases and 200 controls, they concluded that the prediction of OAC by AI is not yet feasible and that expert agreement on the same matter is poor. In contrast, our study demonstrated excellent expert agreement, which may be the result of a defined, preset evaluation criteria system (see Appendix 1).

Our investigation revealed that neither the presence nor the size of periapical inflammation significantly influences the occurrence of oroantral communication (OAC). This finding may be attributable to the preservation of the cortical bone at the sinus base during bone resorption or to local thickening of the Schneiderian membrane induced by inflammation. Both factors potentially diminish the risk of creating a pronounced, direct connection during tooth extraction. Furthermore, disruptions in the basal contour of the maxillary sinus or the presence of root projections in relation to the sinus base did not demonstrate a significant impact.

The results of our study identified several potential indicators on panoramic radiographs predictive of OAC formation. Both decision tree analysis and binary logistic regression revealed significant correlations with two parameters: the distance between the level of the bone and the cementoenamel junction mesially, and the maximal root projection. While the decision tree offers a clinically relevant heuristic for decision-making, it is important to note that the initial branching point (mesial CEJ to marginal bone distance) provides limited interpretive value, as a negative distance may denote either an impacted tooth or a a root remnant. The depth of impaction or, alternatively, the extent of destruction (distance from the bone margin to the CEJ or the most coronal part of the root) may affect the development of OAC. This relationship is corroborated by our binary logistic regression analysis, which indicated a 0.721-fold decrease in OAC risk for every millimeter increase in the mesial distance from the CEJ to the bone. The significance of this measure may extend beyond the relative depth of the tooth/root, suggesting that the removal of a root remnant or impacted tooth may necessitate osteotomy or intensive use of elevators, both of which are likely contributory to OAC formation. These potential contributing factors were beyond the scope of this study. Another significant determinant of OAC, identified by both analytical approaches, was the maximal root projection. A one-millimeter increase in maximal root projection was associated with a 1.22-fold increase in OAC risk, with the decision tree threshold set at 3.3 mm. This finding is consistent with prior research by Madjar et al.[12], who demonstrated that root projection into the sinus is overestimated on panoramic radiographs compared to CBCT, emphasizing the significance of the extent, rather than the mere presence, of root projection.

Sinus recess and mesial or distal angulation of the tooth were also identified as significant factors by the decision tree. The relevance of sinus recess is underscored by Regnstrand et al.[8] who observed that the roots may contact the sinus across a larger surface area, not limited to the socket's most apical portion. Mesial angulation has been highlighted as a risk factor by other studies [6, 20]. It is noteworthy that teeth with distal angulation were relatively infrequent (*n* = 11) in our cohort, suggesting the need for further investigation into their significance. The binary logistic regression analysis also identified the loss of mesial contact as a significant OAC risk factor. The presence of a mesial adjacent tooth was associated with a 0.495-fold reduction in OAC risk. This factor is inherently related to mesial angulation and sinus recess on the mesial side; tooth loss can lead to mesialization and sinus pneumatisation over time. The interplay among these factors adds complexity to the analysis.

Our findings advocate for the consideration of various clinical and radiographic indicators on panoramic radiographs when predicting the likelihood of OAC in association with upper tooth extractions. To the best of our knowledge, this is one of the inaugural studies to evaluate the incidence of OAC following tooth extractions using routine panoramic radiographs, with a particular focus beyond the upper wisdom teeth. Our interrater reliability was good to excellent, validating the effectiveness of our predefined criteria and the reliability of the study's results.

Nonetheless, certain limitations of the study warrant mention. Panoramic radiography only allows for semi-standardized settings, the two-dimensional images lack the anatomical details provided by a three-dimensional CBCT image and distortion also hinders precise linear measurements. Given the retrospective nature of the study, specific clinical data—such as periodontal probing depth surrounding the tooth, tooth mobility, precise localization of OAC within the alveolar socket, or detailed accounts of the instruments used and the difficulty of the extraction procedure—could not be collected. Additionally, the variability in operator technique was not addressed due to the involvement of numerous dentists in the extractions. Sample size was limited by the occurrence of OAC during the study period, a multi-center study with uniform protocol, x-ray device, and setting could yield a significant increase in case numbers.

While CBCT remains the superior imaging modality for predicting OAC during dental extractions, its use is constrained by cost, radiation exposure, and environmental impact. A prospective clinical study incorporating comprehensive preoperative examinations, meticulously documented interventions, and precise measurements taken from well-aligned periapical radiographs using the parallel technique could yield additional valuable data for the prediction of post-extraction OAC. In the absence of CBCT imaging, clinicians relying on PR images can still utilize several warning signs to predict post-extraction OAC.

## Supplementary information

Below is the link to the electronic supplementary material.

Supplementary file1 (DOCX 21 KB)

## Data Availability

All data supporting the findings of this study are available within the paper and its Supplementary Information. No datasets were generated or analysed during the current study.
