# Supplementary material for: Evaluation of clinical and radiographic warning signs for prediction of oroantral communication following tooth extractions
Source: Clin Oral Investig. 2024 Oct 23;28(11):609. doi: 10.1007/s00784-024-06000-x (PMC11499406; doi:10.1007/s00784-024-06000-x)
Supplement: Supplementary file 1 — Supplementary file1 (DOCX 21 KB) [file 784_2024_6000_MOESM1_ESM.docx]

Evaluation Criteria on panoramic radiographic (PR) images

1. Adjacency of Mesial and Distal Teeth: The presence of adjacent teeth mesial and distal to the extracted tooth was recorded.

2. Restorative Status: The presence and type of dental restoration were categorized as absent, present with a single-surface filling, present with a multi-surface filling, or present with a crown.

3. Root Canal Treatment: The radiographic evidence of gutta-percha within the root canals was assessed.

4. Carious Lesions: The presence of caries was noted as absent or present. If present, the extent was further classified as single-surface, multi-surface, or encompassing all surfaces of the crown/complete destruction (radix).

5. Tooth Angulation (based on Winter, 1926 and Archer, 1975): Teeth were classified as having vertical, mesial, or distal angulation.

6. Number of Roots: Teeth were categorized as having a single root or multiple roots.

7. Root Length Mesially and Distally: The distance from the marginal bone's coronal level to the apex was measured on the mesial and distal sides, respectively. In single-rooted teeth, measurements were taken on both sides of the root; in teeth with distinct mesial and distal roots, the respective roots were measured.

8. Impaction Status: Teeth were evaluated as either impacted or not impacted.

9. CEJ to Marginal Bone Distance: The distance from the cementoenamel junction to the coronal level of the marginal bone was measured mesially and distally. In cases where the CEJ was obscured by restorations or severe destruction, the apical portion of the restoration or the most coronal portion of the remaining root was used as a reference point. Note that in cases of impacted teeth and other aforementioned scenarios, this value could be negative.

10. Interdental Distance: Measurements were made at four sites: mesial coronal and distal coronal at the bone level, and mesial apical and distal apical at the base of the maxillary sinus or the most apical point, if the root did not reach the sinus base. In quadrants lacking teeth/roots, the median sagittal suture served as the mesial reference point, and the distal side of the maxillary tuberosity served as the distal reference point.

11. Sinus recess: Pneumatization of the sinus between roots, manifesting as an indentation or elongation on the maxillary sinus base, was recorded as present or absent on the mesial and distal side of the extracted tooth.

12. Root Indentation Mesially and Distally: The distance from the root tip to the sinus base was measured mesially and distally. This parameter reflects the extent of root projection into the sinus. A negative value was assigned if the root did not reach the sinus base.

13. Maximal Root Indentation: This was calculated based on the root that projected farthest into the sinus or was closest to the sinus base if no roots reached it. For a single-rooted tooth, this value would correspond to one of the measures in point 12. For multi-rooted teeth, especially molars with a palatal root projecting furthest into the sinus, this value would be distinct.

An average of the previous three parameters was also computed as the average root indentation.

14. Sinus Base Relation: The positional relationship between the tooth roots and the sinus base was classified as one of the following:

a. Roots do not contact the sinus base.

b. Roots contact the sinus base without projecting further.

c. Roots project into the sinus.

15. Vertical Bone Defect Presence and Depth: Distinct vertical bone loss on the mesial or distal side of the tooth was identified on radiographs. When present, the depth of the defect was measured from the cortical bone level to the base of the defect. If defects were present on both sides, the average of the two depths was calculated.

16. Periapical Defect Presence and Extent: The presence of distinct periapical radiolucency around one or more roots, indicative of chronic periapical inflammation, was noted. When present, the size was measured by its maximum vertical and mesiodistal width. In cases of multiple separated lesions, the lesion with the largest diameter was recorded and used in subsequent analyses.

17. Periapical Lesion to Sinus Base Relationship: This was classified as either:

a. The lesion does not contact the sinus base.

b. The lesion contacts or penetrates the sinus base.

18. Sinus Base Visibility: The continuity of the sinus base around the evaluated tooth was classified as either uninterrupted/visible or interrupted/non-visible.

19. Vertical Bone Width Mesially and Distally: The distance between the cortical bone layers (coronal bone level and base of the sinus) was measured on the mesial and distal sides of the tooth..

Bibliography:

1. Winter GB: Impacted Mandibular Third Molar. St Louis, MO, American Medical Book Co, 1926
2. Archer HW: Oral and Maxillofacial Surgery (ed 5). Philadelphia, PA, WB Saunders, 1975, p 311
